# The Oxoglutarate Binding Site and Regulatory Mechanism Are Conserved in Ammonium Transporter Inhibitors GlnKs from *Methanococcales*

**DOI:** 10.3390/ijms22168631

**Published:** 2021-08-11

**Authors:** Marie-Caroline Müller, Tristan Wagner

**Affiliations:** Microbial Metabolism Research Group, Max Planck Institute for Marine Microbiology, Celsiusstraße 1, 28359 Bremen, Germany; mmueller@mpi-bremen.de

**Keywords:** P_II_-family protein, 2-oxoglutarate, nitrogen regulation, X-ray crystal structure, conformational switch, energy charge, methanogenic archaea, marine thermophile

## Abstract

Protein inhibition is a natural regulatory process to control cellular metabolic fluxes. P_II_-family signal-transducing effectors are in this matter key regulators of the nitrogen metabolism. Their interaction with their various targets is governed by the cellular nitrogen level and the energy charge. Structural studies on GlnK, a P_II_-family inhibitor of the ammonium transporters (Amt), showed that the T-loops responsible for channel obstruction are displaced upon the binding of 2-oxoglutarate, magnesium and ATP in a conserved cleft. However, GlnK from *Methanocaldococcus jannaschii* was shown to bind 2-oxoglutarate on the tip of its T-loop, causing a moderate disruption to GlnK–Amt interaction, raising the question if methanogenic archaea use a singular adaptive strategy. Here we show that membrane fractions of *Methanothermococcus thermolithotrophicus* released GlnKs only in the presence of Mg-ATP and 2-oxoglutarate. This observation led us to structurally characterize the two GlnK isoforms apo or in complex with ligands. Together, our results show that the 2-oxoglutarate binding interface is conserved in GlnKs from *Methanococcales*, including *Methanocaldococcus jannaschii*, emphasizing the importance of a free carboxy-terminal group to facilitate ligand binding and to provoke the shift of the T-loop positions.

## 1. Introduction

P_II_-family proteins are key players in the regulation of nitrogen metabolism in bacteria, archaea and in the plastids of plant/algae [1]. Their functions can include ammonium transport inhibition [2,3], ammonium-fixation regulation by indirectly or directly controlling the glutamine synthetase [4,5] and N_2_-fixation inhibition by interfering with the dinitrogenase reductase [6,7]. P_II_ proteins are not limited by protein level regulation and can control gene transcription by interacting with transcriptional regulators involved in nitrogen assimilation [8,9]. Their known range of action was recently extended to the control of CO_2_-fixation and sensing of fluctuating inorganic carbon levels in cyanobacteria [10,11] and it is expected that such fascinating sensors might also have undescribed roles in nature. Most of P_II_ proteins are regulated by the binding of two effectors: 2-oxoglutarate (2OG), whose concentration depends on the cellular carbon and nitrogen status (Figure S1), and ADP/ATP, which are indicators of the energy charge [12,13]. Because of their regulatory importance, numerous P_II_ protein structures from different organisms were elucidated. All P_II_ proteins known so far show a typical homotrimeric organization [14]. The monomeric unit, of *circa* 11–18 kDa, consists of two α-helices, four β-strands (organized in a double ferredoxin-like fold β1α1β2β3α2β4) and three protruding loops called B, C and T [14]. In the trimeric state, clefts are formed at the dimeric interface through the interaction of the B-loop and the base of the T-loop of one subunit with the C-loop of the adjacent one. These clefts constitute the binding site for ADP/ATP, magnesium (Mg) and 2OG [1,7,14,15]. The T-loop is of greater importance since it has been determined to be essential for the interaction with target proteins. Furthermore, it contains the residue Tyr51, a site of uridylylation in bacteria, which strongly affects target binding [1,16].

Detailed studies highlighted the molecular mode of interaction between the P_II_-family protein GlnK and its target, the ammonium channel Amt. First described in *Escherichia coli* [2,3], crystal structures of GlnK–Amt revealed the intimacy of the interaction. GlnK in complex with ADP binds Amt in a 3:3 ratio where each ordered T-loop is inserted into one of the cytoplasmic exit channels of the Amt trimer. Ammonia transfer through the channel is physically blocked by the GlnK sidechain Arg41 located on the T-loop. While ADP promotes GlnK–Amt complexation, the opposite is the case for the binding of the effectors ATP, Mg and 2OG, which lead to a shift in the T-loop positions further outward from the center of the trimer, precluding an insertion into the Amt exit channels [17]. ATP and Mg are required for 2OG binding. 2OG triggers the conformational rearrangement of the T-loop.

Studies in archaea revealed a well-conserved mode of action and regulation of GlnK homologues [17,18]. However, structural and biochemical investigations by Yildiz et al. on the GlnK–Amt system in the hyperthermophile *Methanocaldococcus jannaschii* suggested that methanogenic archaea have a different type of regulation [19]. They obtained a crystal structure of GlnK_1_ (*Mj*GlnK_1_) in which the 2OG was found to be bound at the top of the T-loop instead of the inter-subunit cleft (Figure S2) [19]. In this scenario, the 2OG attached at the top of the T-loop would prevent GlnK association to Amt and therefore allow ammonium import. Yet, recombinantly expressed *Mj*Amt_1_ and *Mj*GlnK_1_ showed an almost complete dissociation when only ATP and Mg were added while 2OG addition by itself showed moderate dissociation. Since *Methanococcales* are energy extremophiles with a majority of diazotrophs among them [20], a tuning of the GlnK regulation with a reduced effect of 2OG might have occurred to allow a stronger influence of the cellular energy charge compared to the carbon and nitrogen status.

To verify this hypothesis, we studied the behavior of GlnK–Amt in membrane extracts from *Methanothermococcus thermolithotrophicus* (a thermophilic marine methanogen) and performed structural analyses on all present GlnK isoforms (*Mt*GlnK_1_ and *Mt*GlnK_2_) as well as GlnK_1_ from *Methanocaldococcus jannaschii* without a C-terminal tag (*Mj*GlnK_1_^woT^). By combining biochemistry studies with structural analyses, we showed that GlnKs from *Methanococcales* bind 2OG in the same binding site as and with the same mechanism of typical proteins in the P_II_-family.

## 2. Results

### 2.1. 2OG, Mg and ATP Are Required to Disrupt MtGlnKs from the Membrane Fraction

**Figure 1:**
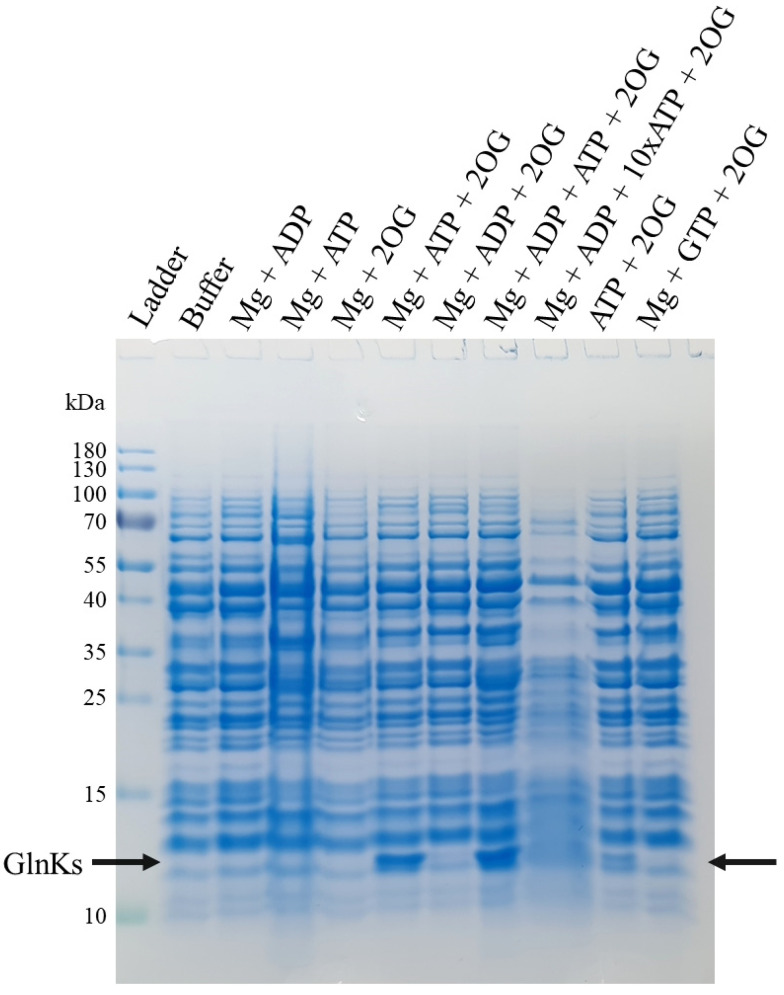
SDS-PAGE of the membrane release experiment with different added ligands. Arrows indicate the position of *Mt*GlnKs on the gel.

Two isoforms of Amt and their respective GlnK regulator are present in the genome of *M. thermolithotrophicus* (Figure S3). They co-localize in the same part of the genome, in contrast to the dispatched organization in *M. jannaschii*. The role of ATP, Mg and 2OG as the physiological triggers to release GlnK from Amt was investigated by using membrane fractions natively isolated from *M. thermolithotrophicus*. During cell lysis, the concentration of the cellular ATP, Mg and 2OG was considered to be sufficiently low to allow the binding of GlnKs to Amt. The membrane resuspension was then washed several times and incubated with different combinations of ATP, ADP, GTP, Mg and 2OG to observe the influence on GlnK release. An ultracentrifugation step was used after incubation to separate unbound proteins, these supernatants were concentrated and passed on SDS PAGE. As shown in Figure 1, a band migrating at circa 12-kDa appeared when ATP, Mg and 2OG were added together. The band was identified by mass spectrometry and mainly contains *Mt*GlnK_1_ and *Mt*GlnK_2_. The peptide counts indicate *Mt*GlnK_2_ as the predominant isoform under this condition with a ratio of one *Mt*GlnK_1_ for two *Mt*GlnK_2_ (see Section 4.3). When only ATP and 2OG were added without Mg, a moderate release of GlnKs was observed that could be explained by residual cations bound to the membrane preparation. ADP or GTP did not disrupt the interaction and a ratio of 1:10 or 1:1 ADP/ATP had no influence on the released amount of *Mt*GlnKs (Figure 1). The observed release of GlnKs from the membrane fraction upon addition of Mg-ATP/2OG fit the established model for GlnK ligand interaction [17,21]. However, they differ from the results obtained with the recombinant Amt_1_ and GlnK_1_ from *M. jannaschii* expressed in *E. coli*, in which the addition of Mg-ATP alone resulted in ~95% of Amt_1_/GlnK_1_ dissociation and 2OG alone resulted in 30% of complex disruption [19].

Such discrepancies between *M. jannaschii* and other studied systems led us to examine the structure of *Mj*GlnK_1_. Compared to the structures of other GlnKs in complex with Mg-ATP and 2OG, the C-terminal His-tag of *Mj*GlnK_1_ seems to interfere with the 2OG binding site (Figure S2) as already laid out by Truan and collaborators [22]. To evaluate the C-terminal extension’s effect on ligand binding, *Mj*GlnK_1_ was produced and crystallized without a tag. Additionally, we performed structural studies on both *Mt*GlnKs isoforms to learn if *Methanococcales* share a conserved ligand-binding site and conformational switch.

### 2.2. MtGlnK_1_ and MtGlnK_2_ Show Remarkable Purification and Crystallization Behaviors

On the way to the crystal structures, we observed astonishing biochemical and crystallization properties of both GlnKs from *M. thermolithotrophicus*. Both N-terminal tagged isoforms were highly expressed and soluble; however, they could not bind on Nickel-NTA resin, probably because of tag hindrance. Since this protein family has been shown to remarkably tolerate high temperatures and to spontaneously refold [23], a boiling step was performed on the soluble cell extract. *Mt*GlnKs tolerated more than 60 min at 70 °C without showing signs of deterioration (Figure S4A). Therefore, this step was used to facilitate their purification. Surprisingly, both isoforms aggregated when placed at 4 °C in a buffer devoid of salts (50 mM Tricine pH 8, 2 mM DTT, Figure S4B). The white flocks were separated from *E. coli* contaminants by centrifugation and the pellet contained a remarkably pure protein preparation (Figure S4C), which could be solubilized by increasing the salinity (e.g., 500 mM NaCl) and the temperature (e.g., 25–70 °C). This treatment maintained the protein solubility and no aggregates were observed during gel filtration (see Section 4.4).

The boiling step was also used to purify *Mj*GlnK_1_^woT^ and the preparation was considerably enriched using this method; however, compared to *Mt*GlnKs, the protein did not precipitate at 4 °C after an overnight incubation and a gel filtration step was performed to further purify the protein (see Section 4.4). *Mt*GlnK_1_ without ligands and *Mt*GlnK_2_ with Mg-ATP/2OG formed macroscale-size crystals in seconds (see Section 4.5). Growth kinetics could be very well controlled by modifying the protein concentration (Figure S4D). Crystals growth could also be controlled via temperature, for instance, it was possible to obtain large usable macro-crystals from micro-crystals by repeating an incubation of the crystallization tray at 46 °C for 3 min followed by incubation at 18 °C (Figure S4E). *Mt*GlnK_1_ and *Mt*GlnK_2,_ crystallized without ligands formed crystals belonging to trigonal and orthorhombic forms that diffracted to a resolution of 1.94 and 2.30 Å, respectively. The *Mt*GlnK_2_ and *Mj*GlnK_1_^woT^ crystals with Mg-ATP/2OG belonging to tetragonal and rhombohedral forms diffracted to an almost atomic resolution of 1.16 Å and 1.20 Å, respectively. This resolution allowed us to reach a high level of detail for the structural analyses.

This one-day two-step purification protocol without the need for column chromatography providing fast crystallization of high-quality diffracting crystals highlights *Mt*GlnKs as accessible protein benchmarks for crystallization and crystallography studies.

### 2.3. Conformational Rearrangement upon Ligand Binding

**Figure 2:**
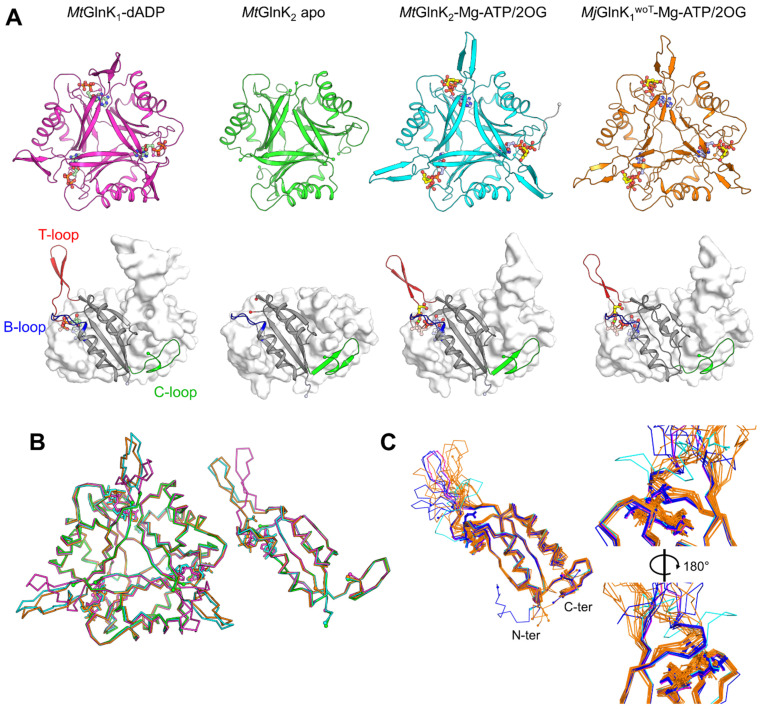
Crystal structures and comparison of the different states of GlnKs from *Methanococcales*. (**A**) Top row: Trimeric GlnK structures are represented in cartoon with ligands in ball and stick. Bottom row: one chain is in cartoon representation while the two others are displayed as white surface. The T-, B- and C-loops are colored in red, blue and green, respectively. Carbon atoms are colored in yellow for 2OG, in pale green for dADP, in pale blue for ATP while nitrogen, oxygen, phosphorus and Mg are colored in blue, red, orange and green, respectively. The N-terminal tag of *Mt*GlnK_2_-Mg-ATP/2OG is colored in light grey and belongs to the symmetry mate. N- and C-termini as well as the cut in the T-loop are shown with spheres. (**B**) Superposition of all obtained structures as trimer (**left**) and monomer (**right**) in ribbon and stick representation. Chains and atoms are colored according to panel A top. (**C**) Superposition of all available P_II_ structures containing ADP or Mg-ATP/2OG are shown in ribbon with ligands in sticks (see Figure S5 for more details). Structures with ADP are colored in orange, structures with Mg-ATP/2OG in blue, *Mj*GlnK_1_-Mg-ATP/2OG with tag (2J9E) in cyan and *Mj*GlnK_1_^woT^-Mg-ATP/2OG in pink. Overall view of the monomer (**left panel**) and close-up of the ligand binding site (**right**).

All obtained structures exhibit the typical trimeric organization and typical fold of the P_II_-family in which β-sheets organize the trigonal core while the α-helices are located at the periphery (Figure 2A, Figure S5), as described in other work [14,24]. The three main loops, T- (residues 36–55), B- (residues 82–89) and C- (residues 102–112) were identified based on their high structural similitude with the GlnK homologue from *A. fulgidus* (Figure 2A). The N-terminal His-tag is only partially observable in the *Mt*GlnK_2_-Mg-ATP/2OG structure and appears to be highly flexible, without interfering with the ligand binding site.

**Figure 3:**
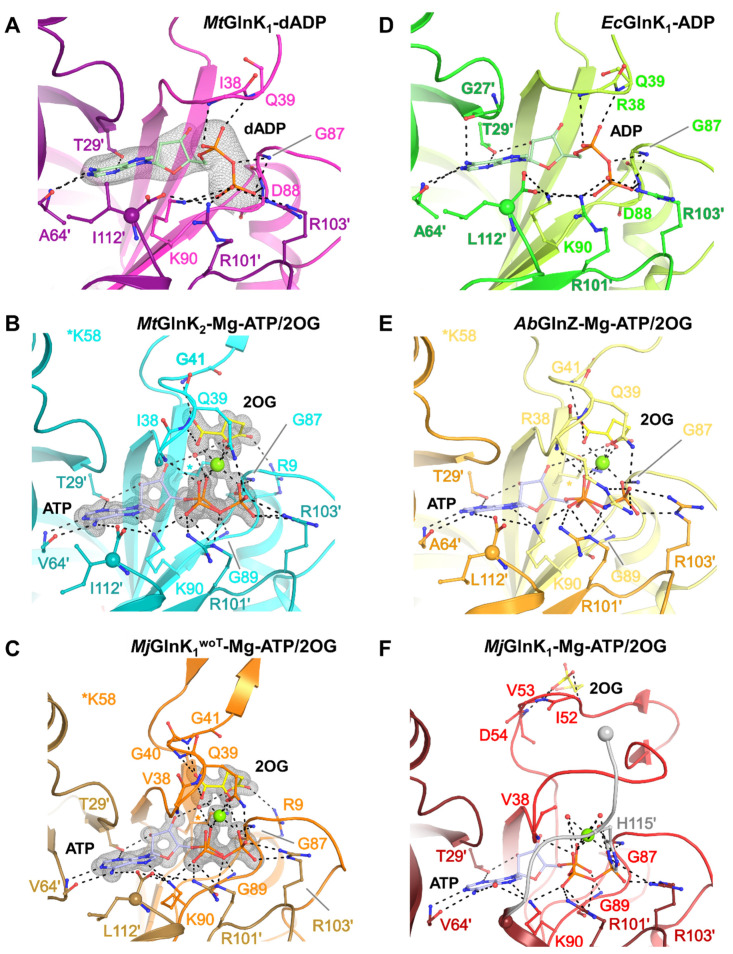
Ligand binding sites. For all panels, the main chain is represented in cartoon. Ligands, main and side chains of the residues participating in ligand binding are shown as ball and stick. Carbon atoms are colored in yellow for 2OG, in pale green for dADP and ADP, in pale blue for ATP while nitrogen, oxygen, phosphorus and Mg are colored in blue, red, orange and green, respectively. C-termini are highlighted by a sphere. Electron density maps (2*F*_o_–*F*_c_) around the ligands are contoured at 2-σ and shown as grey mesh. Polar contacts are indicated by black dashes. (**A**) *Mt*GlnK_1_-dADP, (**B**) *Mt*GlnK_2_-Mg-ATP/2OG, (**C**) *Mj*GlnK_1_^woT^-Mg-ATP/2OG, (**D**) *E. coli* GlnK-ADP (PDB:2NUU), (**E**) *Azospirillum brasilense* GlnZ-Mg-ATP/2OG (PDB:3MHY), (**F**) tagged *Mj*GlnK_1_-Mg-ATP/2OG (PDB:2J9E) with its C-terminal tag colored in light grey.

Unexpectedly, the structure of *Mt*GlnK_1_ crystallized without ligands contains an additional electron density in the cleft surrounded by the three loops. A 2′-deoxyadenosinediphosphate (dADP) molecule was modelled with high confidence (Figure 3A and Figure S6). Since no nucleotide was added during the purification, the metabolite must originate from the *E. coli* cytoplasm and has been retained during the whole purification. dADP is coordinated by a network of hydrogen bonds from Thr29′, Ile38, Gln39, Gly87, Asp88, Lys90, Ala64′, Arg101′ and Arg103′. This state has a similar nucleotide coordination and T-loop positions compared to the *E. coli* AmtB-GlnK structure (Figure S7). We suggest that the presence of a dADP rather than ADP is artefactual and we consider that dADP would not influence the T-loop coordination differently compared to ADP (Figure 3D).

An extra electron density for nucleotides does not exist in the ligand-binding site of the *Mt*GlnK_2_ apo structure and seems to be replaced by water molecules. However, an additional electron density lies between the modelled GlnK_2_ trimers composing the asymmetric unit. The resolution limit, the high twinned fraction (0.39) as well as high translational non-crystallography symmetry (56%) and the poorly defined extra electron density restrained us from placing a model that seems to be an *Mt*GlnK_2_ homodimer.

The T-loops are visible in the structures of *Mt*GlnK_1_-dADP, *Mj*GlnK_1_^woT^-Mg-ATP/2OG and *Mt*GlnK_2_-Mg-ATP/2OG. Modelled T-loops are, however, still more flexible compared to the core of the protein due to their high local B-factors. The T-loop’s tip positions might be influenced due to their participation in the crystal packing (Figure S8). As expected, the T-loop is drastically switching position between the *Mt*GlnK_1_-dADP and the ternary complex structure from *Mt*GlnK_2_-Mg-ATP/2OG and *Mj*GlnK_1_^woT^-Mg-ATP/2OG (Figure 2B), with a maximum deviation of 11.3 Å and 10.7 Å, respectively (measured at the Cα of the Arg47).

Structure comparison of the ternary complexes from *Mt*GlnK_2_-Mg-ATP/2OG and *Mj*GlnK_1_^woT^-Mg-ATP/2OG and structural homologues shows an excellent fit of the T-loop at its base (Figure 2C and Figure S5) and some deviation at the top of the loop which could be due to the crystal contacts. In comparison, the structure of tagged *Mj*GlnK_1_ with Mg-ATP and 2OG shows a striking deviation. This difference likely results from an effect of the tag (Figure S2). Therefore, a close inspection of the ligand-binding site was warranted.

### 2.4. The Free Carboxy-Terminal Is Indirectly Stabilizing 2OG Binding

**Figure 4:**
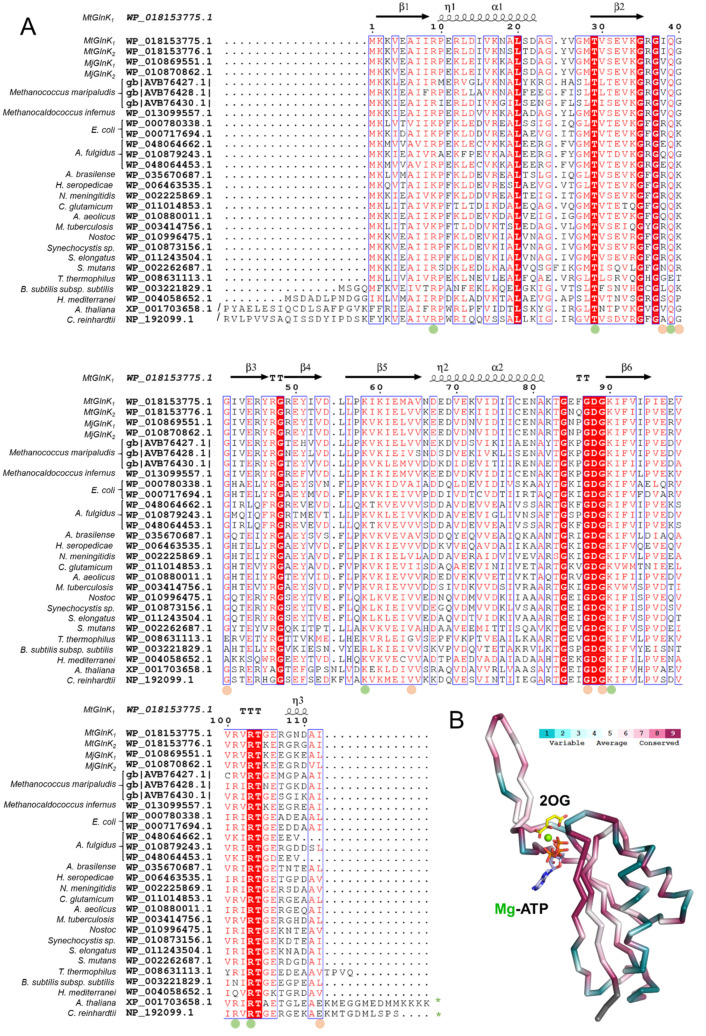
Sequence conservation among structurally characterized P_II_ members with the addition of various *Methanococcales* (see Supplemental materials for the full names). (**A**) Sequences were aligned with Clustal omega and processed with Espript 3.0. *Mt*GlnK_1_ was used as a reference for the secondary structure. Dots indicate residues involved in ligand binding via side chain (green) or main chain (orange). Green stars indicate a C-terminal extension (Q-loop). (**B**) Sequence conservation score superposed on the 3D-structure of *Mt*GlnK_2_-Mg-ATP/2OG monomer processed via the ConSurf Server [25,26]. The conservation is represented as a color gradient, from blue (variable) to dark red (conserved). Nitrogen, oxygen, phosphorus and Mg are colored in blue, red, orange and green, respectively. Carbons of 2OG and ATP are colored yellow and pale blue, respectively.

*Mt*GlnK_2_ and *Mj*GlnK_1_^woT^ structures, co-crystallized with Mg-ATP and 2OG, offered a very clear picture of the ligand coordination due to their very high-resolution (see Section 4.6). Both structures show an almost identical coordination of the ligands. The adenine part of ATP is flanked by the phenyl group of Phe92 and the whole molecule is coordinated by hydrogen bonds from Thr29′, Ile38, Gln39, Gly87, Lys90, Val64′, Arg101′ and Arg103′ (residues are based on *Mt*GlnK_2_, Figure 3B,C). For the 2OG, the keto group is coordinated by the backbone of Gln39, Gly41, while the Arg9 and Lys58 coordinate the carboxy group via salt bridges. The carboxy group has an equivalent position as the Gln39 side chain in the *Mt*GlnK_1_-dADP structure. Mg plays an important role as a bridging agent, binding the three phosphate groups of the ATP, the keto acid group of the 2OG and the side chain of Gln39 that closes the hexa-coordination (Figure 3B,C). The residues involved in ligand-binding are conserved to a certain extent throughout the P_II_-family (Figure 4). The residues binding ligands by their main chain can be variable and the ones binding through the side chains are all well conserved.

A superposition of each three different chains of the tagged and untagged *Mj*GlnK_1_ with Mg-ATP and 2OG shows a different position of the C-loop and the effect of the tag (Figure S9). In *Mj*GlnK_1_^woT^, the carboxy-terminus is coordinating the adenine ring and the Lys90, the latter adequately engages a salt bridge with the β-phosphate of the ATP. The carboxy-terminus is itself locked by the side chain of the Ile via hydrophobic interaction. Such a critical position seems to be very well conserved in GlnK-subfamily as observed in *A. brasilense* GlnZ [22] (Figure 3E). Therefore, we propose that the absence of the carboxy-terminal provokes a slight displacement of the Lys90 that shifts the coordination of the β-phosphate group. Moreover, the addition of the poly-histidine tag displaces the Mg which cannot bind the 2OG anymore (Figure 3F). The absence of an appropriate 2OG coordination blocks the correct positioning of the T-loops and led to its aberrant conformation (Figure S9).

## 3. Discussion

P_II_-family proteins represent a large class of regulators found across the three domains of life [27] and are involved in maintaining the cellular carbon/nitrogen balance. They share a high structural conservation and ligand binding mode that pose them as an ideal target for drug design with a focus on nitrogen regulation. New exciting discoveries pointed out that P_II_-family proteins are not restrained to the cellular nitrogen control but also carbon-fluxes in cyanobacteria [10,11]. Therefore, with expanding availability of P_II_ protein sequences, biochemical characterizations are needed to elucidate yet undiscovered physiological functions, targets and regulation systems. The T-loop is the essential component for target interaction and can vary in length and composition depending on the system. It contains key residues targeted for post-translational modification in some organisms and undergoes conformational changes upon ADP/ATP-Mg-2OG binding. While 2OG reflects the cellular nitrogen/carbon status, the ADP/ATP balance represents the energy level of the cell, an important factor for methanogenic archaea, considered to be energy extremophiles. Therefore, it could be possible that nitrogen regulation in these microorganisms may be regulated exclusively by the ADP/ATP balance instead of the cellular 2OG concentration. This hypothesis appears to be supported by previous work on *M. jannaschii* where 2OG was found to have a reduced effect on the Amt_1_/GlnK_1_ disruption caused by a different 2OG binding mode. Such exception among P_II_-family proteins is not unique, as GlnK_2_ from *A. fulgidus* and SbtB from cyanobacteria were not found to bind 2OG [11,28].

The results described here point out that the unconventional behavior observed in *M. jannaschii* is most likely due to the C-terminal His-tag of the *Mj*GlnK_1_ construct. The tag hides the terminal carboxy group and indirectly displaces the loop coordinating the 2OG, generating an altered position of the T-loop. As proposed before by Truan and coworkers [22], the carboxy-terminal group is of importance for ligand binding stabilization. The tag in the N-terminal position seems less consequential, as we did not see any differences in ligand coordination between *Mt*GlnK_2_-Mg-ATP/2OG and *Mj*GlnK_1_^woT^-Mg-ATP/2OG structures.

Interestingly, a free C-terminal carboxy group at an equivalent position is not critical for ligand binding as shown in other P_II_-family proteins that contain a C-terminal extension. For instance, in *Chlamydomonas reinhardtii* and *Arabidopsis thaliana* the C-terminus is extended by the so-called Q-loop able to bind glutamine [29]. In the structure of the P_II_-family protein from *C. reinhardtii* containing Mg-ATP and 2OG, the end of the C-loop and the whole Q-loops are flexible and not modelled. Compared to GlnK, this protein harbors an N-terminal extension folded close to the C-loop that might be an adaptation to the loss of the free carboxy group in the C-terminus.

Our studies also highlighted that *Mt*GlnK_1_ is able to bind a deoxynucleotide without provoking further structural changes (Figure 3A and Figures S6 and S7). The dADP might come from an artefact during the boiling procedure and its physiological relevance should be investigated in the future. Intriguingly, it seems that *Mt*GlnKs switch is not under the dependency of the energy charge since addition of ADP to the membrane experiment did not inhibit the release. However, we cannot exclude a possible effect of AMP to displace the equilibrium toward an activation of GlnKs.

Both *Mt*GlnK isoforms were detected in the membrane dissociation experiment and one can wonder why this organism would have two versions of Amt and GlnK especially if these isoforms vary barely in sequence and structure. Solid-supported membrane electrophysiology on Amts from *A. fulgidus* proved that even with low divergence sequences, functional differences exist between the three different isoforms [30]. The associated GlnK regulators might be tuned up to also recognize their specific transporters. Therefore, we tentatively modelled *Mt*AmtB_1_ (WP_018153774) and *Mt*AmtB_2_ (WP_018153777) based on an *E. coli* structure (PDB: 2NUU) to visualize the surface charges of the binding interface between *Mt*GlnKs and their transporters (Figure S10). Since electrostatic charges are very similar for both *Mt*GlnKs we cannot conclude on the specificity towards the two modelled AmtB isoforms. However, our model supports that in the dADP-bound state, the T-loop could in theory block the NH_3_-channel after slight repositioning as shown in *E. coli* structures. Contrarily, the fixed T-loop positions in the Mg-ATP/2OG bound state prevent insertion into the Amt channel, as demonstrated in previous studies [17].

## 4. Materials and Methods

### 4.1. Cultivation of M. thermolithotrophicus

*M. thermolithotrophicus* strain DSM 2095 was ordered from the Deutsche Sammlung von Mikroorganismen und Zellkulturen (DSMZ, Leibniz, Germany) and was cultivated at 65 °C in a minimal mineral medium. For one liter of medium the compounds listed in Table 1 were subsequently dissolved in 750 mL of deionized H_2_O (dH_2_O). The pH was set to 6 with NaOH pellets. The media was filled up to a final volume of 1 L by the addition of dH_2_O.

Trace element composition: A 100-fold-concentrated trace element solution was prepared by first dissolving 1.36 g nitrilotriacetic acid (7.1 mM) in 800 mL dH_2_O under magnetic stirring. The pH was adjusted to 6.2 by adding NaOH pellets. The components listed in Table 2 were successively added and the solution was filled up to a final volume of 1 L with dH_2_O.

*M. thermolithotrophicus* was grown under strict anaerobic condition in a 10 L fermenter containing 7 L of medium gassed with up to 50 kPa H_2_/CO_2_ and 50 kPa N_2_ under 500 rpm stirring at 65 °C. The fermenter was inoculated with 600 mL preculture cultivated in the same medium. The cells were grown until late exponential phase (OD_600_ of 1.35) and then immediately transferred in an anaerobic tent (N_2_/CO_2_ atmosphere at a ratio of 90:10). Cells were harvested by anaerobic centrifugation for 30 min at 4500× *g* at 18 °C. 7 L of culture yielded 19.5 g of cells (wet weight). The cell pellet was transferred in a sealed bottle, gassed with 30 kPa with N_2_, flash frozen in liquid N_2_ and stored at −80 °C.

### 4.2. Membrane Experiment, Binding Assay

To investigate the interaction behavior of Amt-GlnK, membrane fractions were isolated natively. Approximately 7 g of *M. thermolithotrophicus* cells were lysed by the addition of 50 mL lysis buffer (50 mM Tricine/NaOH pH 8 and 2 mM dithiothreitol, DTT) followed by sonication (five cycles of 10 s sonication at ~65% intensity with 20 s breaks, probe MS73, Bandelin SONOPULS, Berlin, Germany,) and centrifuged at 45,000× *g* for 45 min at 20 °C. The cell lysate was transferred and centrifuged at 150,000× *g* for 1 h at 25 °C in an ultracentrifuge (Beckman Coulter Life Sciences, Krefeld, Germany, L-70) to isolate the membrane fraction. The supernatant was discarded, and the membrane fraction was resuspended in the washing buffer (50 mM Tris/HCl pH 7.6, 500 mM NaCl and 2 mM DTT) followed by ultracentrifugation at 150,000× *g* for 1 h at 25 °C. This washing procedure was performed four times. The pellet was resuspended in the lysis buffer and split evenly in 10 aliquots of 10 mL. The following metabolites were then added: (1) no effector; (2) 2 mM MgCl_2_, 2 mM ADP; (3) 2 mM MgCl_2_, 2 mM ATP; (4) 2 mM MgCl_2_, 10 mM 2OG (oxoglutarate sodium salt); (5) 2 mM MgCl_2_, 2 mM ATP, 10 mM 2OG; (6) 2 mM MgCl_2_, 2 mM ADP, 10 mM 2OG; (7) 2 mM MgCl_2_, 2 mM ADP, 2 mM ATP, 10 mM 2OG; (8) 2 mM MgCl_2_, 2mM ADP, 20 mM ATP, 10 mM 2OG; (9) 2 mM ATP, 10 mM 2OG; (10) 2 mM MgCl_2_, 2 mM GTP, 10 mM 2OG. All conditions were incubated for 30 min at room temperature, then centrifuged at 150,000× *g* for 1 h at 25 °C. The supernatant was concentrated to 100 µL in a 10-kDa cut-off concentrator (Vivaspin^®^ 20, Sartorius, Göttingen, Germany) and analyzed by SDS-PAGE (Anode buffer: 100 mM Tris base and 22.5 mM HCl, pH 8.9 and Cathode buffer: 100 mM Tris base, 100 mM Tricine and 0.1% sodium dodecyl sulfate (SDS) pH 8.25) for 90 min at 90 Volts. The gel was stained with InstantBlue^TM^ (Expedeon, Cambridgeshire, UK).

### 4.3. Protein Identification by Mass Spectrometry

Selected Coomassie stained bands were excised, and the proteins within were reduced by DTT, alkylated by iodoacetamide and digested overnight with trypsin. The resulting peptides were extracted twice by 5% formic acid and the extracts were pooled together and dried down. The samples were then reconstituted in 25 µL of 5% formic acid containing 5 fmol/μL retention time standard peptides (Pierce, Rockford, IL, USA) and 5 µL were taken for LC-MS/MS analysis.

LC-MS/MS analysis was performed by MS Facility at the MPI-CBG Dresden, Germany, on a nano-UPLC Ultimate 3000 interfaced on-line to an Orbitrap HF hybrid mass spectrometer (both Thermo Fischer Scientific, Bremen, Germany). The UPLC system was equipped with Acclaim™ PepMap™ 100, 75 µm × 2 cm trapping column (Thermo Fischer Scientific, Bremen, Germany) and 50 cm μPac micro Pillar Array separating column (PharmaFluidics, Ghent, Belgium). Peptides were separated using 75 min linear gradients 0% to 65% of acetonitrile (solvent A—0.1% formic acid in water, solvent B—0.1% formic acid in neat acetonitrile). Spectra were acquired using Top20 data-dependent acquisition method, precursor *m*/*z* range was 350–1600 and dynamic exclusion time was 15 s. The lock-mass function was set to recalibrate MS1 scans using the background ion (Si(CH_3_)_2_O)_6_ at *m*/*z* 445.12. Acquired spectra were searched by MASCOT software (version 2.2.04) against *M. thermolithotrophicus* protein sequences in NCBI database (1799 entries, September 2020) and genome (6-frames translation). Mass tolerance was set to 5 ppm and 0.025 Da for precursor and fragment ions respectively; two miscleavages; variable modifications—cysteine carbamidomethyl, propionamide; methionine oxidized. The results were evaluated by Scaffold software (Proteome Software version 4.11.0, Portland, USA), minimum number of matched peptides was set to two; peptide and protein false discovery rate (FDR) was below 1%.

The total spectrum counts for *Mt*GlnK_1_ and *Mt*GlnK_2_ were 346 and 784, respectively for the Mg-ATP/2OG condition (from the gel of Figure 1); 420 and 869 for the Mg-ATP-ADP/2OG condition; 244 and 705 for the ATP/2OG condition.

### 4.4. GlnKs Expression and Purification

*Mt*GlnK_1_, *Mt*GlnK_2_ sequences originate from *M. thermolithotrophicus* strain DSM 2095 and *Mj*GlnK_1_ sequence originates from *M. jannaschii* strain DSM 2661. DNA coding sequences were codon optimized (refer to the supplementary data for the sequences) and synthesized by Genscript (Leiden, Netherlands). For *Mt*GlnK_1_ and *Mt*GlnK_2_, NdeI/BamHI restriction sites were added in the 5′- and 3′-extremities, respectively. Both synthetic genes were cloned in a pET-28a(+) vector by Genscript.

For *Mj*GlnK_1_, NdeI and XhoI restriction sites were added in the 5′- and 3′-extremities, respectively. A stop codon was also added upstream of the XhoI site in 3′. The DNA synthesized was cloned in the expression vector pET-24b(+) at the NdeI and XhoI restriction enzyme digestion sites by Genscript.

Each construct was expressed in *Escherichia coli* strain BL21(DE3) cultivated in Lysogeny Broth supplemented with a final concentration of 50 µg/mL kanamycin. The cultures were incubated at 37 °C, being shaken, until an OD_600_ of 0.6–0.8 was reached. Induction was performed by adding Isopropyl ß-D-1-thiogalactopyranoside at a final concentration of 0.75 mM and the cells were incubated for another hour at 37 °C, being shaken. The cells were harvested by centrifugation at 5000× *g* for 20 min at 21 °C. Subsequently, the cell pellet was washed in phosphate buffered saline solution and centrifuged for 20 min at 4000× *g* at room temperature. The washed cell pellets were flash-frozen in liquid nitrogen.

The frozen cell pellets were thawed and resuspended in the lysis buffer (50 mM Tricine/NaOH pH 8, 2 mM DTT) at a ratio of 4 mL lysis buffer per gram of wet weight. The cells were disrupted by sonication (10 cycles of 20 s at 70% intensity with 30 s breaks, probe KE 76, Bandelin SONOPULS, Berlin, Germany) on ice. The lysate was centrifuged at 45,000× *g* at room temperature for 40 min. The supernatant was then heated up to 70 °C for 30 min followed by centrifugation for 20 min at 45,000× *g*. The supernatant was filtered through 0.2 µm (Sartorius, Göttingen, Germany) and placed at 4 °C overnight, standing.

For *Mt*GlnK_1_ and *Mt*GlnK_2_, a white precipitate appeared overnight and the sample was centrifuged at 45,000× *g* for 30 min at 4 °C. The pellets, corresponding to the protein of interest, were resuspended in 4 mL of gel filtration buffer (25 mM Tris/HCl pH 7.6, 10% glycerol, 2 mM DTT, 500 mM NaCl) and the aggregates were dissolved by heating at 70 °C for 15 min followed by centrifugation 13,000× *g* for 5 min RT. The supernatants were immediately injected on a HiLoad^®^ 16/600 Superdex^®^ 200 pg (GE Healthcare, Freiburg, Germany). The protein was eluted at a flow rate of 1 mL/min at room temperature. *Mt*GlnK_1_ eluted as a single gaussian peak at 82 mL, from which the fractions of interest were pooled. *Mt*GlnK_2_ major peak eluted at 86.6 mL. Both pools were concentrated with a 10-kDa cut-off concentrator (Vivaspin^®^ 20, Sartorius, Göttingen, Germany) at room temperature and centrifuged at 13,000× *g* for 5 min to remove aggregates.

For *Mj*GlnK_1_, no precipitate appeared after the overnight incubation at 4 °C. The sample was filtered through 0.2 µm (Sartorius, Göttingen, Germany) and immediately injected on a HiLoad 16/600 Superdex 200pg (GE Healthcare, Freiburg, Germany). The sample eluted at a flow rate of 1 mL/min in the gel filtration buffer and four peaks were observable at elution volumes of 62, 66, 73 and 80 mL. The four different pools were concentrated with a 10-kDa cut-off concentrator (Vivaspin^®^ 20, Sartorius, Göttingen, Germany) at room temperature and centrifuged at 13,000× *g* for 5 min to remove aggregates.

All purification steps were systematically checked by SDS-PAGE. The protein concentration was determined via Bradford assay. All GlnKs passed on SDS-PAGE exhibited an extra band at 25-kDa, in addition to the expected monomeric molecular weight (circa 12 kDa), as observed in other GlnK studies [31].

### 4.5. Crystallization

All purified proteins were immediately crystallized at 18 °C. Initial screening was performed by using the sitting drop method on 96-Well MRC 2-Drop Crystallization Plates in polystyrene (SWISSCI, Neuheim, Switzerland) containing 90 µL of crystallization solution in the reservoir. 0.6 µL sample with 0.6 µL crystallization solution were spotted. For all three GlnKs, the protein sample was crystallized with and without 2 mM ATP, 2 mM 2OG and 2 mM MgCl_2_. Since *Mt*GlnK_1_ and *Mt*GlnK_2_ with ligands aggregated strongly at 18 °C, the protein sample was first heated to 70 °C and centrifuged at 13,000× *g* for 5 min at room temperature to remove remaining aggregates before spotting.

*Mt*GlnK_1_ at a concentration of 33 mg/mL co-crystallized with ligands generated few crystals but none of them diffracted to an exploitable resolution. However, hexagonal rods instantly appeared for *Mt*GlnK_1_ at 33 mg/mL without ligands in 35% *w*/*v* Pentaerythritol propoxylate (17/8 PO/OH), 100 mM MES; pH 6.5 and 200 mM ammonium sulfate.

*Mt*GlnK_2_ at a concentration of 30 mg/mL without ligand crystallized as small squares in 35% *w*/*v* Pentaerythritol ethoxylate (15/4 EO/OH) and 100 mM HEPES pH 7.5. When *Mt*GlnK_2_ at 30 mg/mL was crystallized with ligands, high-quality shard shaped crystals appeared after minutes in 25% *w*/*v* Pentaerythritol ethoxylate (3/4 EO/OH) and 100 mM MES pH 6.5.

As stated above, four pools of purified *Mj*GlnK_1_ were obtained after gel filtration. The best crystals were obtained from the pool containing the protein eluted at 66 mL on the gel filtration. This pool, concentrated to 11.2 mg/mL, was co-crystallized with ligands and high-quality cube-shaped crystals appeared overnight in 20% *w*/*v* PEG 3350, 100 mM Bis-Tris propane pH 8.5 and 200 mM sodium nitrate.

### 4.6. X-Ray Crystallography Data Collection, Refinement and Analyses

*Mj*GlnK_1_ crystals were soaked in their crystallization solutions supplemented with 20% *v*/*v* glycerol as a cryo-protectant prior to being frozen in liquid nitrogen. *Mt*GlnK_2_ crystals with ligands were soaked in the crystallization solution supplemented with 60 mM Tb-Xo4 (Molecular Dimensions, Sheffield, UK) for 3 min 30 s prior to being frozen in liquid nitrogen. The Tb-Xo4 had no effect on the diffraction quality and was not detected in the electron density.

X-ray diffraction was collected at 100 K at the Synchrotron Source optimisée de lumière d’énergie intermédiaire du LURE (SOLEIL), PROXIMA-1 beamline; the Swiss Light Source (SLS), X06DA and X10SA beamlines and at PETRA-III, P11 beamline. The best datasets were obtained at the Swiss Light Source (SLS), X06DA and X10SA beamlines.

Data were processed and scaled with *autoPROC* (Version 1.0.5, Global Phasing Limited, Cambridge, UK) [32]. The structures were solved by molecular replacement with phaser from the *PHENIX* package (Version 1.19.2-4158) [33], using *Mj*GlnK_1_ (PDB 2J9D) as a template. All models were then manually rebuilt with *COOT* (Version 0.8.9.2) [34] and further refined with *PHENIX*. *Mt*GlnK_2_ and *Mj*GlnK_1_ with ligands were refined by considering all atoms anisotropic. *Mt*GlnK_2_ apo was refined with applied translational-liberation screw and twinning refinement, considering a twin fraction of 0.39 with the following twin law: h, k, -l. *Mt*GlnK_1_-dADP was also refined by applying translational-liberation screw. For all models, hydrogens were added in riding position for the last refinement cycles except for *Mt*GlnK_2_ apo. Hydrogens were omitted for the final deposited model.

All models were validated through the MolProbity server (http://molprobity.biochem.duke.edu, accessed on 8 July 2021) [35]. Data collection and refinement statistics, as well as PDB identification codes for the deposited models and structure factors are listed in Table 3.

### 4.7. Structural Analyses

Sequence alignment was performed with Clustal Omega (https://www.ebi.ac.uk/Tools/msa/clustalo/, accessed on 6 July 2021) [36] and analysis was performed with Espript (Version 3.0) [37]. The homology modelling of AmtB_1_ and AmtB_2_ from *Methanothermococcus thermolithotrophicus* based on *E. coli* structure (PDB 2NUU) was performed by the Phyre2 server (http://www.sbg.bio.ic.ac.uk/phyre2/html/page.cgi?id=index, accessed on 6 July 2021) [38]. All figures of protein structure were generated with PyMOL (Version Open-Source PyMOL™ 1.8.x) (Schrödinger, LLC). Sequence conservation score superposed to the *Mt*GlnK_2_-Mg-ATP/2OG structure was performed with the ConSurf server (https://consurf.tau.ac.il/credits.php, accessed on 6 July 2021) [25,26].

## 5. Conclusions

Our work emphasized the structural conservation among GlnKs and the importance of the free carboxy group at the C-terminal to optimize ligand coordination. Therefore, adding a tag at this position should be avoided in future studies on the close members of this protein family to avoid unwanted artefacts.

While our results show that GlnKs from *Methanococcales* do not structurally and mechanistically differ from previously characterized GlnKs, another group of P_II_ proteins present in diazotrophic methanogens might have different properties. These two genes coding for NifI_1_ and NifI_2_ are always co-located within the nitrogenase operon and regulate N_2_-fixation [7]. They are phylogenetically different from GlnKs and biochemical characterization suggested unique traits regarding their oligomerization. Future studies will be necessary to reveal whether NifI is a structural exception within the very well conserved P_II_-family.

## Figures and Tables

**Table 1:** Medium components for *M. thermolithotrophicus* cultivation.

Compound	Amount	Final Molarity
K_2_HPO_4_	55.7 mg	0.32 mM
KH_2_PO_4_	55.8 mg	0.41 mM
KCl	1 g	13.4 mM
NaCl	25.13 g	430 mM
NaHCO_3_	0.81 g	10 mM
CaCl_2_·2H_2_O	367.5 mg	2.5 mM
MgCl_2_·6H_2_O	7.725 g	38 mM
NH_4_Cl	1.18 g	22.06 mM
Fe(NH_4_)_2_(SO_4_)_2_·12H_2_O	29.92 mg	0.031 mM
Nitrilotriacetic acid	61.16 mg	0.32 mM
2 mM Na_2_SeO_3_·5H_2_O stock	10 µL	0.02 µM
Na_2_WO_4_·2H_2_O	3.3 mg	0.01 mM
Na_2_MoO_4_·2H_2_O	2.42 mg	0.01 mM
MES	9.76 g	50 mM
Na_2_SO_4_	1.42 g	10 mM
1.5 mM resazurin	1 mL	0.0015 mM
Trace element solution	10 mL	

**Table 2:** Trace element solution.

Compound	Amount	Final Molarity
MnCl_2_·6H_2_O	91.4 mg	0.45 mM
FeCl_3_·6H_2_O	183.3 mg	0.68 mM
CaCl_2_·2H_2_O	60.26 mg	0.76 mM
CoCl_2_·6H_2_O	180.8 mg	0.76 mM
ZnCl_2_	90 mg	0.66 mM
CuSO_4_·5H_2_O	35.21 mg	0.14 mM
Na_2_MoO_4_·2H_2_O	46 mg	0.19 mM
NiCl_2_·6H_2_O	90 mg	0.38 mM
VCl_3_	30 mg	0.19 mM

**Table 3:** X-ray crystallographic data and refinement statistics.

	*Mt*GlnK_1_ with dADP	*Mt*GlnK_2_ apo	*Mt*GlnK_2_ with ATP, Mg and 2OG	*Mj*GlnK_1_ with ATP, Mg and 2OG
**Data collection**				
Synchrotron source	SLS, PXIII	SLS, PXIII	SLS, PXIII	SLS, PXIII
Wavelength (Å)	1.00003	0.99187	0.99985	0.99187
Space group	*P*321	*P*2_1_2_1_2	*P*4_3_2_1_2	*H*32
Resolution (Å)	75.60–1.94 (2.15–1.94)	73.16–2.30 (2.40–2.30)	57.33–1.16 (1.26–1.16)	44.53–1.20 (1.24–1.20)
Cell dimensions				
a, b, c (Å)	87.296, 87.296, 46.010	103.461, 103.460, 178.491	58.238, 58.238, 229.304	89.053, 89.053, 98.059
α, β, γ (°)	90, 90, 120	90, 90, 90	90, 90, 90	90, 90, 120
R_merge_(%) ^a^	5.3 (171.1)	13.3 (115.1)	4.9 (64.3)	5.7 (120.9)
R_pim_ (%) ^a^	1.3 (40.3)	5.5 (50.8)	1.0 (28.6)	2.0 (44.3)
CC_1/2_ ^a^	0.999 (0.773)	0.998 (0.671)	0.999 (0.774)	0.999 (0.642)
I/σ*_I_* ^a^	27.3 (1.8)	10.2 (1.5)	29.1 (1.7)	17.8 (1.7)
Spherical completeness ^a^	66.3 (12.7)	82.1 (35)	78.1 (17.7)	67.8 (34.6)
Ellipsoidal completeness ^a^	89.1 (60.2)	96.2 (81.3)	93.2 (51.7)	89.8 (99.6)
Redundancy ^a^	19.3 (18.6)	6.7 (6.0)	21.6 (5.2)	9.4 (8.3)
Nr. unique reflections ^a^	10,108 (502)	70,360 (3519)	108,153 (5406)	31,673 (1585)
**Refinement**				
Resolution (Å)	39.30–1.94	73.16–2.30	57.33–1.16	44.53–1.20
Number of reflections	9995	70,326	108,153	31,668
R_work_/R_free_ ^b^ (%)	19.66/22.57	22.15/24.45	11.68/13.66	13.11/15.54
Number of atoms				
Protein	882	9066	2788	953
Ligands/ions	28	13	145	52
Solvent	23	104	516	178
Mean B-value (Å^2^)	62.0	37.0	20.0	21.0
Molprobity clash score, all atoms	1.63	3.89	0.67	0
Ramachandran plot				
Favored regions (%)	100	98.75	100	100
Outlier regions (%)	0	0	0	0
rmsd ^c^ bond lengths (Å)	0.013	0.006	0.014	0.012
rmsd ^c^ bond angles (°)	1.454	0.888	1.592	1.565
**PDB ID code**	7P4V	7P4Y	7P50	7P52

^a^ Values relative to the highest resolution shell are within parentheses. ^b^ R_free_ was calculated as the R_work_ for 5% of the reflections that were not included in the refinement. ^c^ rmsd, root mean square deviation.

## Data Availability

The structures were deposited in the protein data bank under the following ID codes: 7P4V for *Mt*GlnK_1_-dADP, 7P4Y for *Mt*GlnK_2_ apo form, 7P50 for *Mt*GlnK_2_-Mg-ATP/2OG and 7P52 for *Mj*GlnK_1_-Mg-ATP/2OG.

## Supplementary Materials

The following are available online at https://www.mdpi.com/article/10.3390/ijms22168631/s1.
