# Supplementary material for: The Oxoglutarate Binding Site and Regulatory Mechanism Are Conserved in Ammonium Transporter Inhibitors GlnKs from Methanococcales
Source: Int J Mol Sci. 2021 Aug 11;22(16):8631. doi: 10.3390/ijms22168631 (PMC8395244; doi:10.3390/ijms22168631)
Supplement: Supplementary file 1 [file ijms-22-08631-s001.zip › ijms-1322057-supplementary.pdf]

The oxoglutarate binding site and regulatory mechanism are conserved in ammonium transporter inhibitors GlnKs from *Methanococcales*.

Marie-Caroline Müller and Tristan Wagner*

*To whom correspondence may be addressed. Email: twagner@mpi-bremen.de

Supplementary files.

Codon optimized sequences of GlnKs.

List of the full name organisms used in Figure 4.

Figure S1. GlnKs physiological function and mode of action in *Methanococcales*.

Figure S2. Differences in the 2OG binding mode between tagged *Mj*GlnK$_1$ and structural homologues

Figure S3. Overview of the *amt* and *glnK* genes in the *Methanothermococcus thermolithotrophicus* and *Methanocaldococcus jannaschii* genomes.

Figure S4. Two-step purification and controlled crystallization of *Mt*GlnKs.

Figure S5. Superposition of all P$_{II}$-family proteins structures containing ADP or Mg-ATP/2OG.

Figure S6. Omit map of the dADP in *Mt*GlnK$_1$ structure.

Figure S7. Superposition of the dADP-*Mt*GlnK$_1$ structure with the GlnK-Amt complex from *E. coli*.

Figure S8. Crystal packing and T-loop interaction.

Figure S9. Superposition of the *Mj*GlnK$_1^{woT}$ and tagged *Mj*GlnK$_1$.

Figure S10. *Mt*AmtB modelling and electrostatic charge surface interaction with *Mt*GlnKs.

Codon optimized sequences of GlnKs. Restriction sites are highlighted in bold and added stop codon is underlined:

*Mt*GlnK$_1$ (WP_018153775.1)

CATATGAAGAAAGTGGAGGCGATCATTCGTCCGGAACGTCTGGACATCGTGAAGAA
CAGCCTGACCGATGCGGGTTACGTTGGCATGACCGTGAGCGAGGTTAAAGGTCGTG
GCATCCAGGGTGGCATTGTGGAGCGTTACCGTGGTCGTGAATATACCGTTGACCTGC
TGCCGAAGATCAAAATTGAACTGGTGGTTAAGGAAGAGGACGTGGAGAAAATCATT
GATATCATTTGCGAAAACGCGAAGACCGGCAACCAAGGTGATGGCAAAGTGTTCAT
CATTCCGGTTGAGGAAGTGGTTCGTGTTCGTACCAAGGAGCGTGGTCGTGGCGCGAT
TTAATGA**GGATCC**

*Mt*GlnK$_2$ (WP_018153776.1)

CATATGAAAAAAGTAGAAGCCATAATAAGGCCAGAGAGATTAGATATTGTTAAAA
ATTCCCTAACTGATGCTGGTTATGTGGGAATGACCGTTAGTGAAGTTAAAGGAAGAG
GTATCCAAGGGGGAATTGTAGAAAGATACAGAGGAAGAGAATACACAGTTGATCTA
CTTCCAAAAATAAAAATTGAACTTGTAGTTAAGGAAGAAGATGTGGAAAAAATAAT
AGACATAATCTGTGAAAATGCCAAAACTGGAAACCAAGGGGATGGGAAAGTGTTTA
TCATACCTGTTGAAGAGGTTGTAAGGGTAAGAACCAAAGAAAGAGGAAGAGGGGC
AATTTAATGA**GGATCC**

*Mj*GlnK$_1$ (NCBI Reference Sequence: WP_010869551.1)

CATATGAAGAAAGTGGAGGCGATCATTCGTCCGGAGAAGCTGGAAATCGTGAAGA
AAGCGCTGAGCGACGCGGGTTACGTTGGCATGACCGTGAGCGAAGTTAAAGGTCGT
GGCGTGCAGGGTGGCATTGTTGAGCGTTACCGTGGTCGTGAATATATCGTGGATCTG
ATTCCGAAGGTTAAAATCGAGCTGGTTGTGAAGGAAGAGGACGTGGATAACGTTAT
TGACATCATTTGCGAAAACGCGCGTACCGGTAACCCGGGTGATGGCAAAATCTTCGT
GATTCCGGTTGAGCGTGTGGTTCGTGTTCGTACCAAGGAAGAGGGTAAAGAAGCGC
TGTGA**CTCGAG**

List of the full name organisms used in Figure 4.

WP_018153775.1 P-II family nitrogen regulator GlnK1 [*Methanothermococcus thermolithotrophicus*], WP_018153776.1 P-II family nitrogen regulator GlnK2 [*Methanothermococcus thermolithotrophicus*], WP_010869551.1 P-II family nitrogen regulator [*Methanocaldococcus jannaschii*], WP_010870862.1 P-II family nitrogen regulator [*Methanocaldococcus jannaschii*],gb|AVB76427.1|:1-112 Nitrogen regulatory protein P-II [*Methanococcus maripaludis*], gb|AVB76428.1|:1-112 Nitrogen regulatory protein P-II [*Methanococcus maripaludis*], gb|AVB76430.1|:1-112 Nitrogen regulatory protein P-II [*Methanococcus maripaludis*], WP_013099557.1 P-II family nitrogen regulator [*Methanocaldococcus infernus*], WP_000780338.1 MULTISPECIES: P-II family nitrogen regulator [*Enterobacteriaceae*], WP_000717694.1 MULTISPECIES: nitrogen regulatory protein P-II [Gammaproteobacteria], WP_048064662.1 P-II family nitrogen regulator [*Archaeoglobus fulgidus*], WP_010879243.1 P-II family nitrogen regulator [*Archaeoglobus fulgidus*], WP_048064453.1 P-II family nitrogen regulator [*Archaeoglobus fulgidus*], WP_035670687.1 MULTISPECIES: P-II family nitrogen regulator [*Azospirillum*], WP_006463535.1 MULTISPECIES: P-II family nitrogen regulator [*Herbaspirillum*], WP_002225869.1 P-II family nitrogen regulator [*Neisseria meningitidis*], WP_011014853.1 P-II family nitrogen regulator GlnK [*Corynebacterium glutamicum*], WP_010880011.1 P-II family nitrogen regulator [*Aquifex aeolicus*], WP_003414756.1 MULTISPECIES: nitrogen regulatory protein P-II [Corynebacteriales], WP_010996475.1 MULTISPECIES: P-II family nitrogen regulator [Nostocaceae], WP_010873156.1 MULTISPECIES: P-II family nitrogen regulator [unclassified *Synechocystis*], WP_011243504.1 MULTISPECIES: P-II family nitrogen regulator [*Synechococcus*], WP_002262687.1 P-II family nitrogen regulator [*Streptococcus mutans*], WP_008631113.1 MULTISPECIES: P-II family nitrogen regulator [*Thermus*], WP_003221829.1 MULTISPECIES: P-II family nitrogen regulator [*Bacillaceae*], WP_004058652.1 P-II family nitrogen regulator [*Haloferax mediterranei*], XP_001703658.1 nitrogen regulatory protein PII [*Chlamydomonas reinhardtii*], NP_192099.1 nitrogen regulatory P-II-like protein [*Arabidopsis thaliana*]

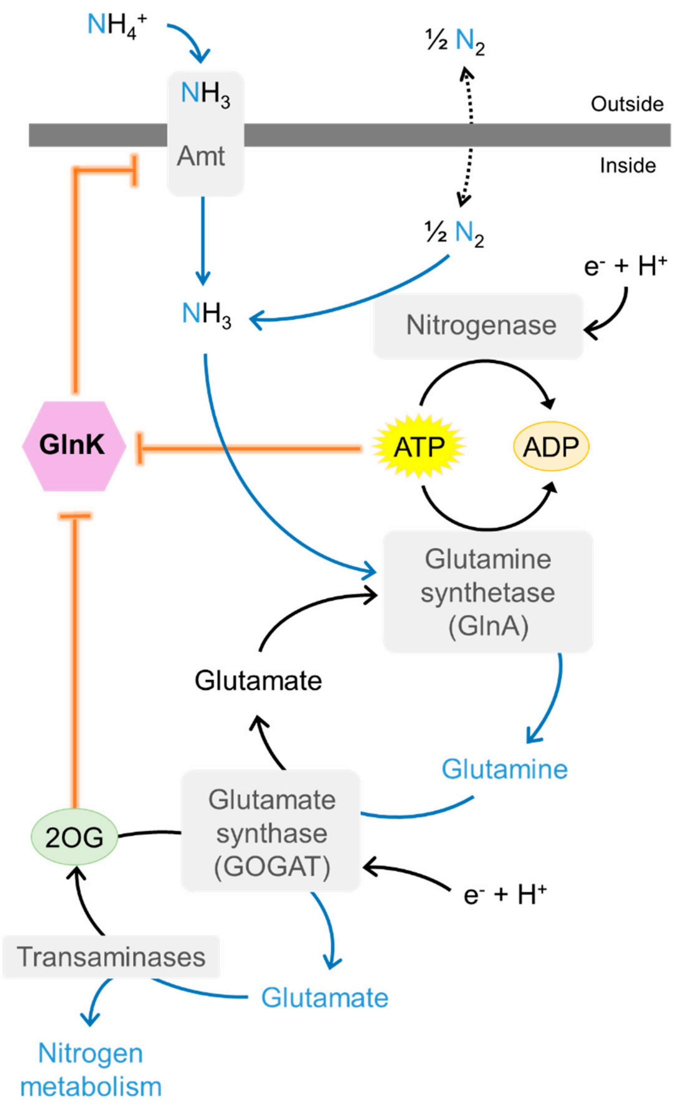

Figure S1. GlnKs´ physiological function and mode of action in *Methanococcales*. Proposed nitrogen-assimilation scheme in diazotrophic *Methanococcales* and its regulation via GlnK, including ATP and 2-oxoglutarate (2OG) as indicators of energy charge and cellular nitrogen state. Orange lines indicate inhibition. Amt has been proposed to channel NH_3 instead of NH_4^+ as discussed in the work of Conroy *et al.*, reference number 2 in the main text.

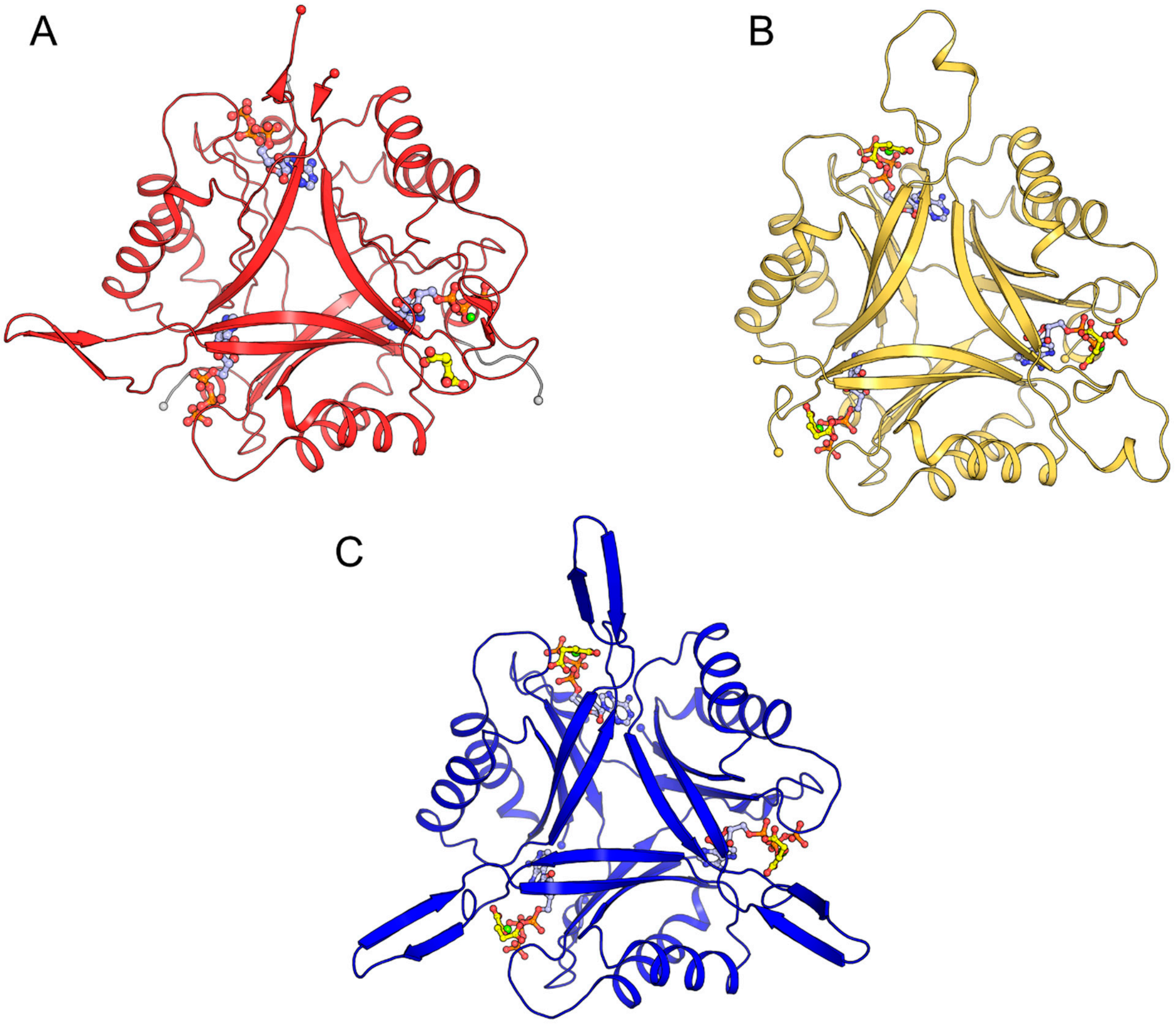

Figure S2. Differences in the 2OG binding mode between tagged *Mj*GlnK$_1$ and structural homologues. GlnKs with bound Mg-ATP/2OG are represented in cartoon with their ligands shown as balls and sticks. N- and C-termini are represented as balls. 2OG carbons are coloured in yellow, ATP carbons in light blue, magnesium in green and nitrogen, oxygen and phosphorus are coloured in blue, red, and orange, respectively. (A) *Mj*GlnK$_1$ PDB code: 2J9E. The C-terminal extension is shown in white. (B) GlnZ from *Azospirillum brasilense* PDB code: 3MHY. (C) GlnK$_3$ from *Archaeoglobus fulgidus* PDB code: 3TA2.

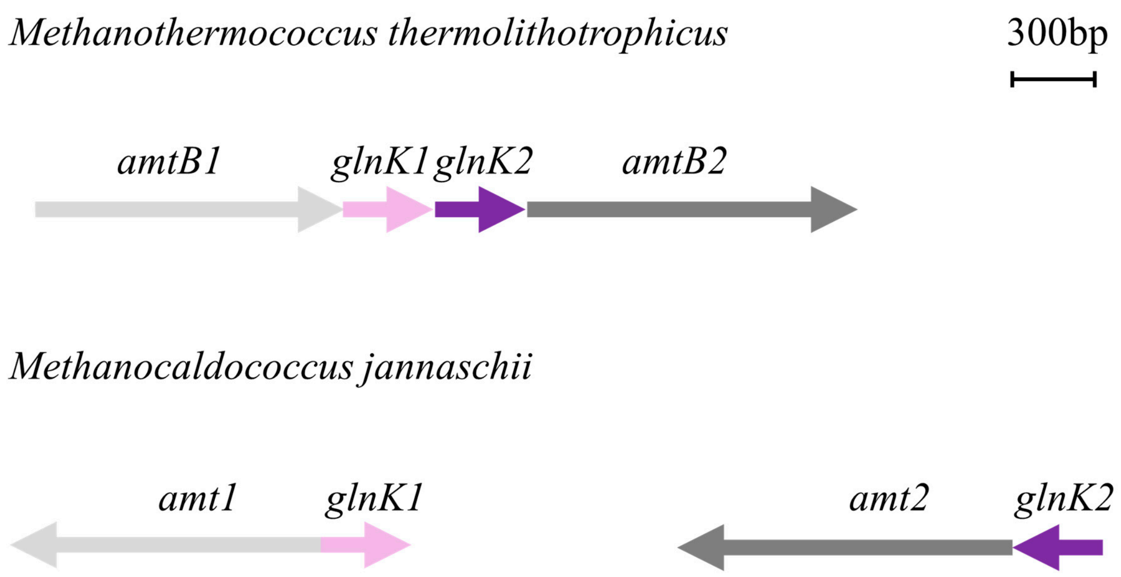

Figure S3. Overview of the *amt* and *glnK* genes in the *Methanothermococcus thermolithotrophicus* and *Methanocaldococcus jannaschii* genomes. Genes are indicated as arrows with isoforms in different shades. Length in base pairs (bp) indicated by a black bar. The two isoforms of *amt* and *glnK* in *M. jannaschii* are not co-localized in the genome.

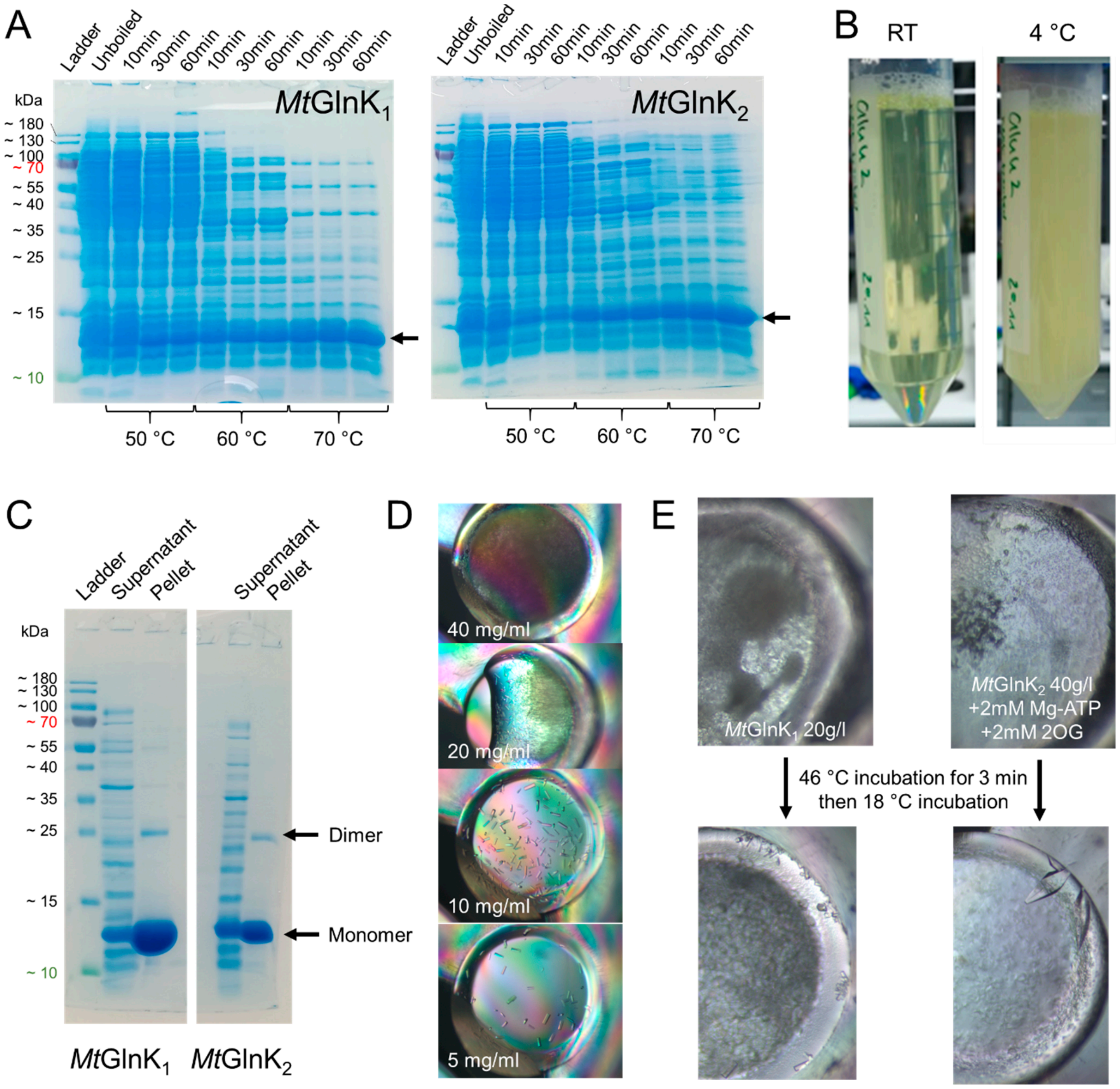

Figure S4. Two-step purification and controlled crystallization of *Mt*GlnKs. (A) Boiling experiment of *Escherichia coli* soluble fractions with overexpressed *Mt*GlnKs. Arrows point to the positions of *Mt*GlnK$_1$ (left panel) and *Mt*GlnK$_2$ (right panel). (B) *Mt*GlnKs behaviour at different temperatures. While the soluble fraction obtained after the 70 °C boiling step treatment appeared soluble at room temperature (left), the sample strongly aggregated after an overnight incubation at 4 °C (right). (C) SDS-PAGE of the protein fraction from the soluble part and the pellet separated by centrifugation of the 4 °C incubated sample shown in B. (D) *Mt*GlnK$_1$ crystallization without ligands at different protein concentrations. (E) *Mt*GlnK$_1$ without ligands (left) and *Mt*GlnK$_2$ with ligands (right) crystals before and after incubation at 46 °C for 3 min.

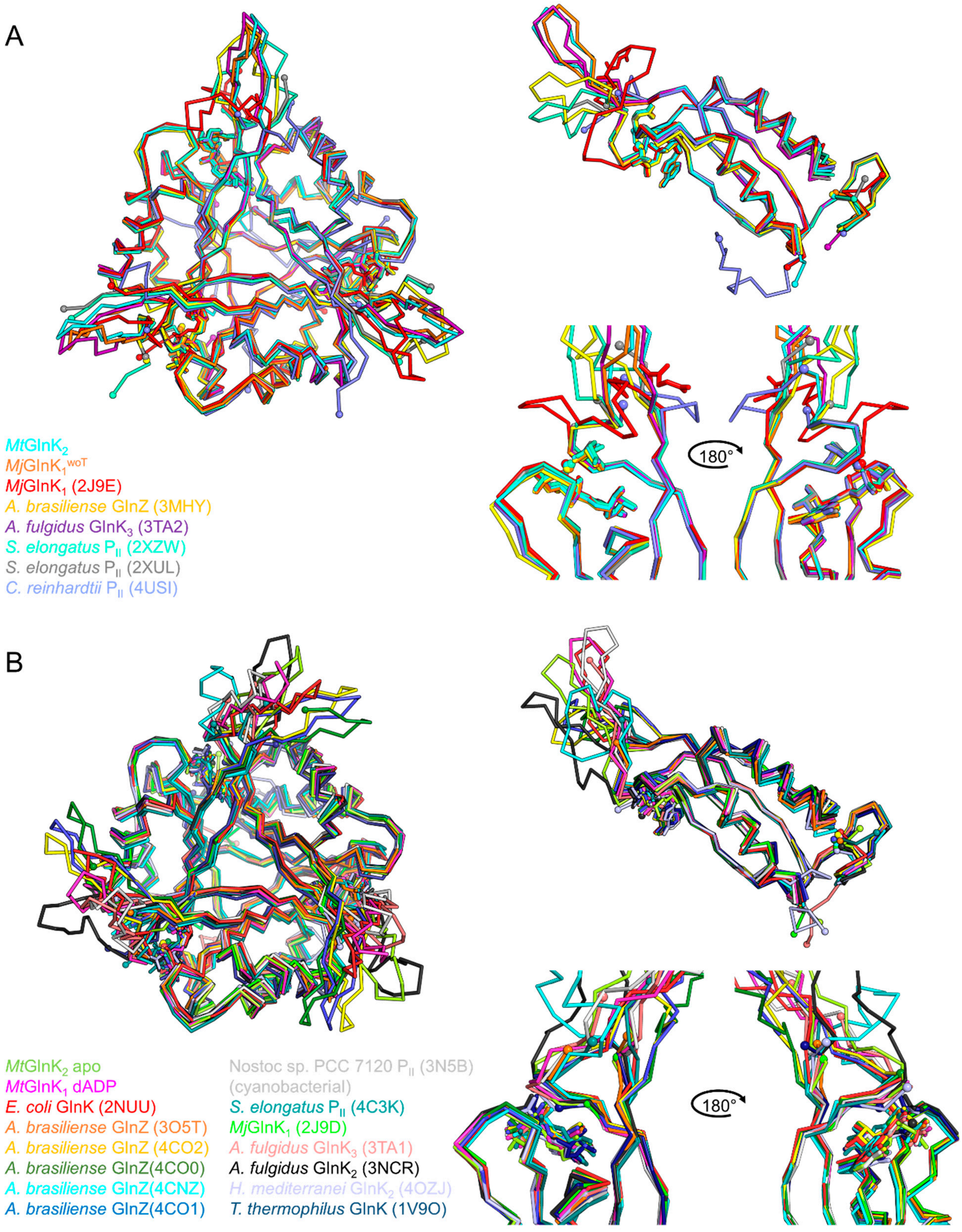
A
MtGlnK$_2$
MjGlnK$_1^{woT}$
MjGlnK$_1$ (2J9E)
A. brasiliense GlnZ (3MHY)
A. fulgidus GlnK$_3$ (3TA2)
S. elongatus P$_{II}$ (2XZW)
S. elongatus P$_{II}$ (2XUL)
C. reinhardtii P$_{II}$ (4USI)
180°
B
MtGlnK$_2$ apo
MtGlnK$_1$ dADP
E. coli GlnK (2NUU)
A. brasiliense GlnZ (3O5T)
A. brasiliense GlnZ (4CO2)
A. brasiliense GlnZ(4CO0)
A. brasiliense GlnZ(4CNZ)
A. brasiliense GlnZ(4CO1)
Nostoc sp. PCC 7120 P$_{II}$ (3N5B) (cyanobacterial)
S. elongatus P$_{II}$ (4C3K)
MjGlnK$_1$ (2J9D)
A. fulgidus GlnK$_3$ (3TA1)
A. fulgidus GlnK$_2$ (3NCR)
H. mediterranei GlnK$_2$ (4OZJ)
T. thermophilus GlnK (1V9O)
180°

Figure S5. Superposition of all P_{II}-family protein structures containing Mg-ATP/2OG or ADP. (A) Superposition of all available GlnK structures with Mg-ATP and 2OG. (B) Superposition of *Mt*GlnK$_2$ apo, *Mt*GlnK$_1$-dADP and all available GlnK structures with ADP. Protein backbones are shown in ribbon representation and ligands in stick representation. N- and C-termini as well as cut T-loops are represented as balls. Trimer (left panel), monomer (top right) and close up of the ligand binding site (bottom right).

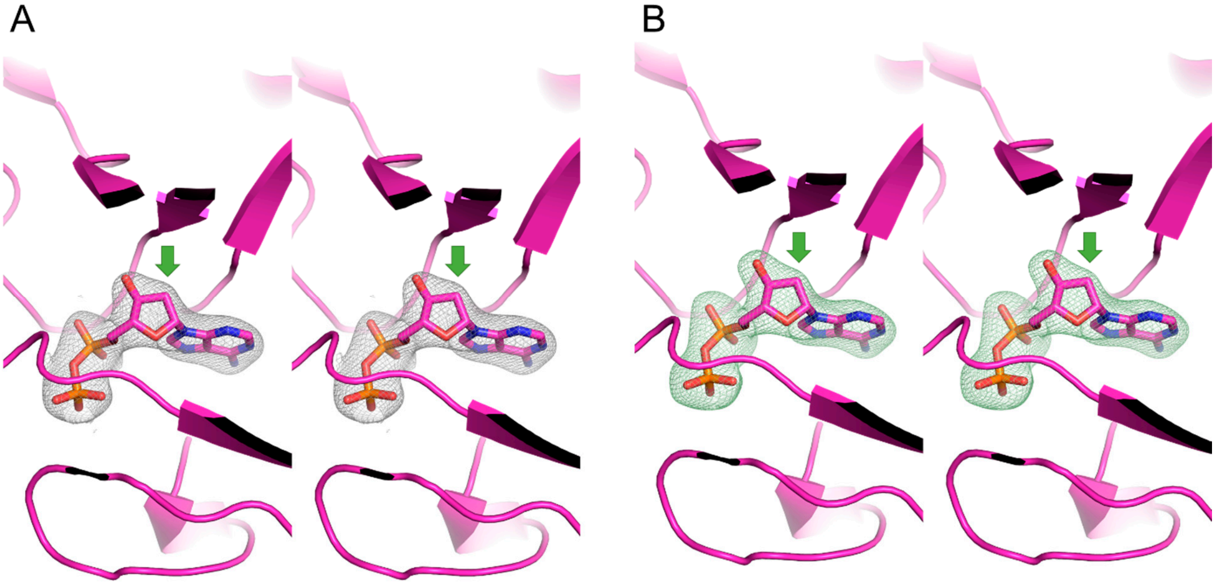

Figure S6. Stereo view of the omit and difference maps of the dADP in the *Mt*GlnK$_1$ structure. (A) The omit map in grey mesh is contoured at 1-σ. (B) The difference map F_o-F_c, is shown as a green mesh and contoured at 3-σ. Arrows indicate the position of the absent hydroxyl group.

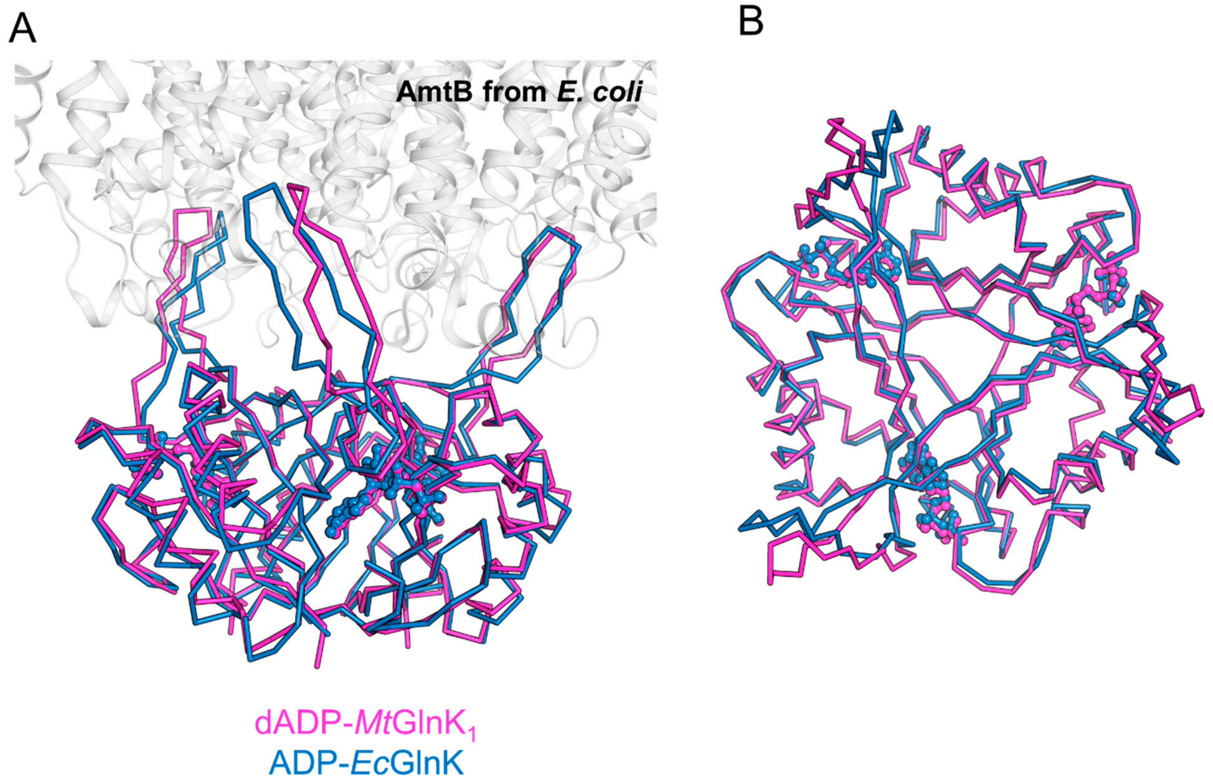

Figure S7. Superposition of the *Mt*GlnK1–dADP structure with the GlnK-Amt complex from *E. coli*. (A) Side view superposition. (B) Top view superposition. *Mt*GlnK1-dADP (pink) with GlnK from *E.coli* (blue) (PDB: 2NUU) are represented as ribbon with ligands as ball and stick. Amt from *E.coli* is shown as white cartoon.

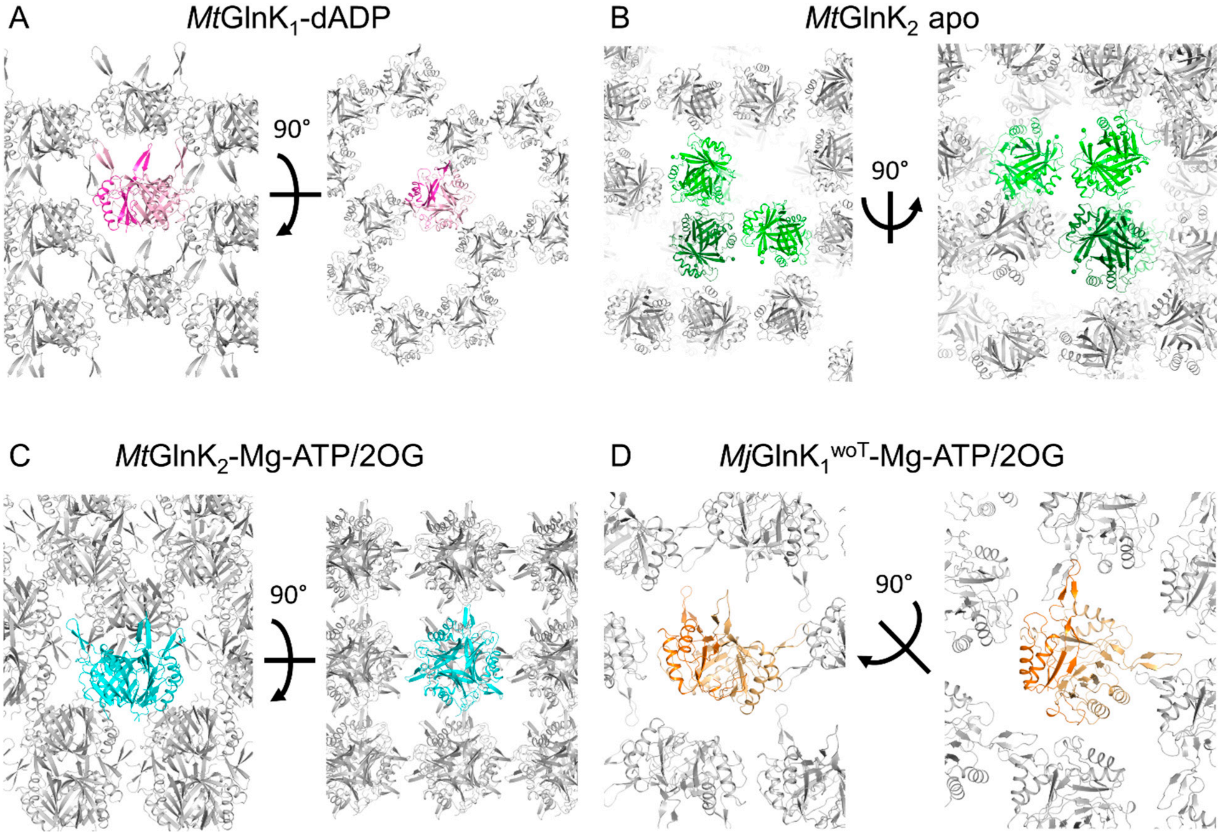

Figure S8. Crystal packing and T-loop interactions. (A) Crystal packing of *Mt*GlnK$_1$-dADP showing the asymmetric unit in pink and the biological unit in light pink. (B) Crystal packing of *Mt*GlnK$_2$ apo showing the asymmetric unit in green and one biological unit in dark green. (C) Crystal packing of *Mt*GlnK$_2$-Mg-ATP/2OG showing the asymmetric unit and biological unit in cyan. (D) Crystal packing of *Mj*GlnK$_1^{woT}$-Mg-ATP/2OG showing the asymmetric unit in orange and the biological unit in light orange.

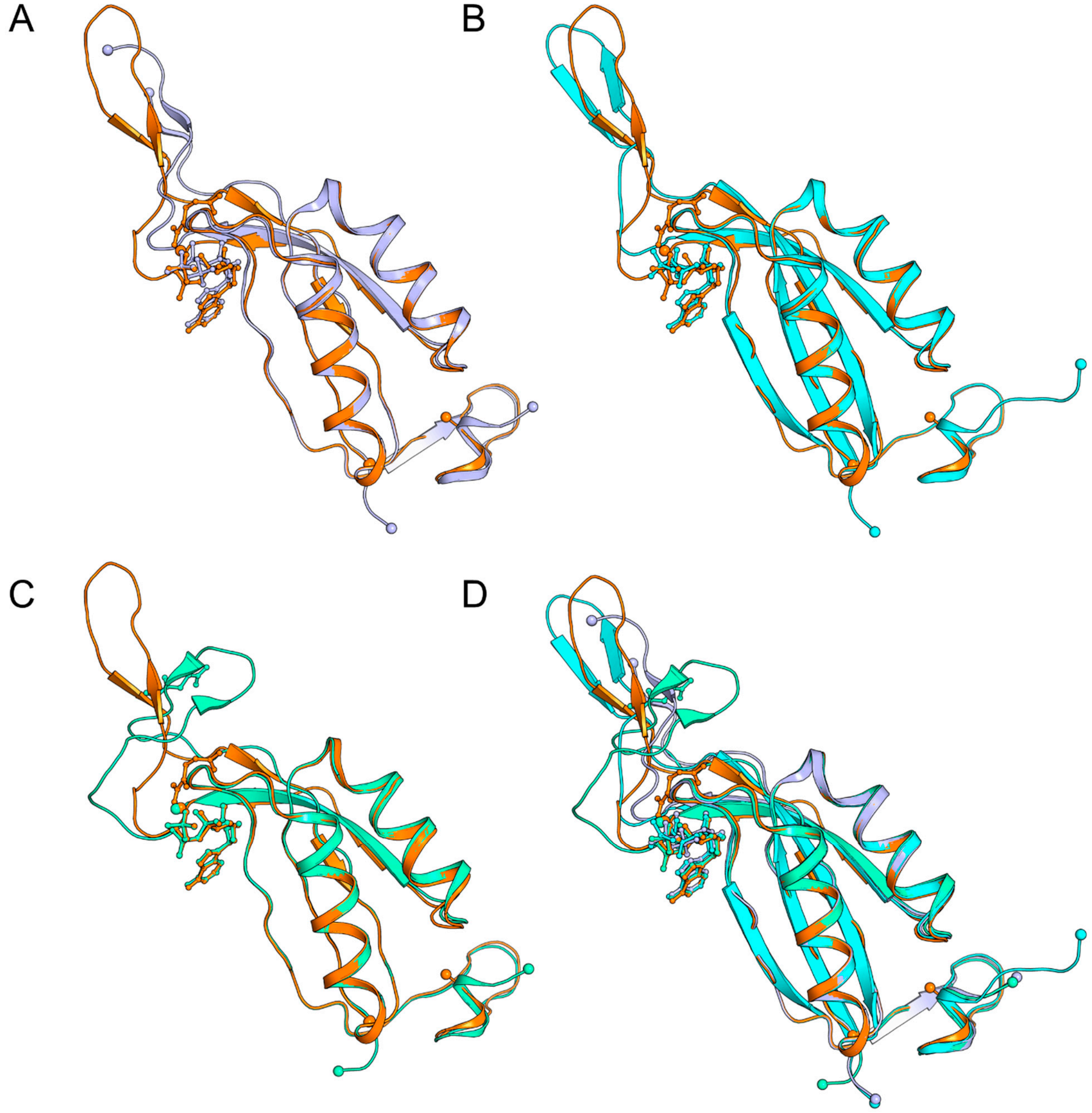

Figure S9. Superposition of *Mj*GlnK$_1^{woT}$ and tagged *Mj*GlnK$_1$. (A) Superposition of *Mj*GlnK$_1^{woT}$ (in orange) with chain A (in light blue) of tagged *Mj*GlnK$_1$ (PDB:2J9E). (B) Superposition of *Mj*GlnK$_1^{woT}$ with chain B (in cyan) of tagged *Mj*GlnK$_1$. (C) Superposition of *Mj*GlnK$_1^{woT}$ with chain C (in light green) of tagged *Mj*GlnK$_1$. (D) Superposition of *Mj*GlnK$_1^{woT}$ with all chains of tagged *Mj*GlnK$_1$.

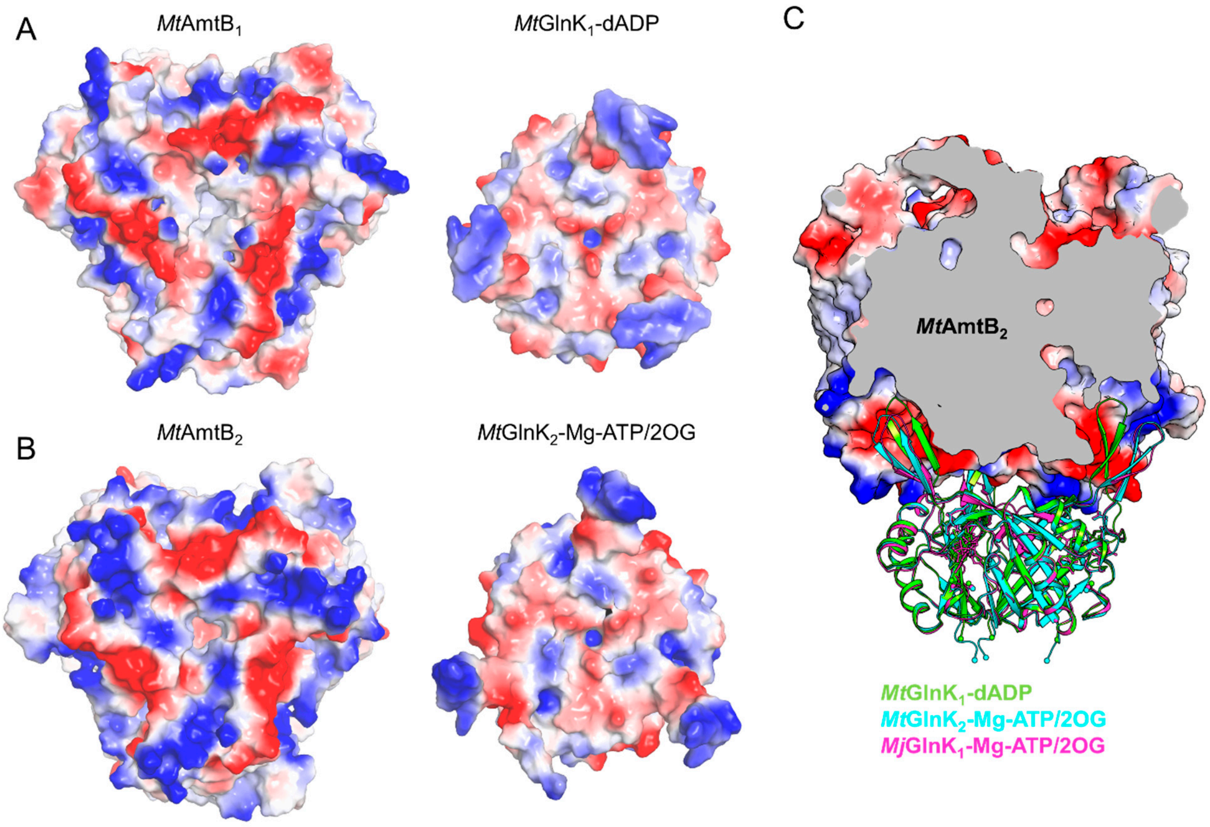

Figure S10. Modelled structures and electrostatic charge surface of *Mt*AmtB isoforms and their predicted interactions with *Mt*GlnKs. *Mt*AmtBs have been modelled based on Amt from *E. coli* (see Materials and methods). The predicted interactions between *Mt*AmtBs and *Mt*GlnKs are based on the *E. coli* model (2NUU). Modelled surface charges are coloured from red (acidic) to blue (basic). (A) Electrostatic surface of the modelled *Mt*AmtB$_1$ (left) and *Mt*GlnK$_1$-dADP (right). (B) Electrostatic surface of the modelled *Mt*AmtB$_2$ (left) and *Mt*GlnK$_2$-Mg-ATP/2OG (right). (C) Cut-through view of modelled *Mt*AmtB$_2$ with *Mt*GlnK$_1$-dADP (green), *Mt*GlnK$_2$-Mg-ATP/2OG (cyan) and *Mj*GlnK$_1^{woT}$-Mg-ATP/2OG (magenta) in cartoon representation.
